# Attitudes and Practice of Health Care Providers Toward Cancer Screening: A Cross-sectional Multicenter Study, Saudi Arabia

**DOI:** 10.1007/s44197-022-00056-2

**Published:** 2022-08-30

**Authors:** Gasmelseed Y. Ahmed, Abbas Al Mutair, Shahinaz Bashir, Rommel Acunin, Nora Al Aljabr, Rasha Alnumari, Ghina Alarab, Siddig Mohamed Hussein, Chandni Saha, Lamiaa H. Al-Jamea, Alexander Woodman, Eman Almusalami

**Affiliations:** 1 College of Medicine and Health Sciences, Almanagil University, Almanagil, Sudan; 2 Research Center Almoosa specialist hospital, Alahsa, Saudi Arabia; 3 School of Nursing, Wollongong University, Wollongong, Australia; 4 Almoosa College of Health Sciences, Alahsa, Saudi Arabia; 5 Nursing Department, Princess Nourah Bint Abdul Rahman University, Riyadh, Saudi Arabia; 6 King Abdullah International Medical Research Center (KAIMRC), Al-Ahsa, Saudi Arabia; 7 King Abdullah International Medical Research Center (KAIMRC), Dammam, Saudi Arabia; 8 Almoosa Specialist Hospital, Alahsa, Saudi Arabia; 9 Vice Deanship of Postgraduate studies and Research, Prince Sultan Military College of Health Sciences, Dhahran, Saudi Arabia

**Keywords:** Cancer, Screening, Health care provider, Knowledge, Attitudes, Practices, Prevention

## Abstract

**Background:**

Screening is a cancer prevention measure for groups who are asymptomatic, and diagnosis is a medical test for groups who are symptomatic. The occupational privilege of health care providers (HCPs) is expected to play a positive role in cancer screening practices. Therefore, this study aimed to assess perceptions and personal attitudes of HCPs regarding their decision to screen for cancer in the Eastern Province of Saudi Arabia.

**Design:**

A cross-sectional multicenter survey study was conducted. A well-designed and validated questionnaire was distributed to the HCPs at three tertiary hospitals in the Eastern Province of Saudi Arabia.

**Results:**

Out of 900 health care providers who received the questionnaire, 372 completed it. Two-thirds, 247 (66.4%) of them were nurses and the rest were physicians and the mean age was 34.1 ± 7.1 years. Regardless of gender, profession, or age, the overall rate of belief in the importance of regular cancer screening was high; 91.4%. The number of participants who did not screen for colonoscopy was significantly higher than those who screened. The number of females in the age group of between 45 and 54 years who screened with mammography was significantly higher than non-screened. In a similar way, male HCPs above 54 years who got themselves screened for Prostate-Specific Antigen (PSA) were significantly higher than those who did not.

**Conclusions:**

Findings of the current research and existing evidence specifically for the Saudi community indicated a need to raise awareness, emphasizing the role of HCPs in motivating themselves, their families, and their patients to implement various cancer screening programs.

## Background

In 2020, estimated 19.3 million new cancer cases and nearly 10.0 million cancer deaths have been reported [1]. The most common cancer was female breast cancer followed by lung cancer, and then colon and rectum cancer [2]. Saudi Arabia was estimated to have between 23,782.8 to 66,899.8 new cancer cases at 2020, and the mortality rate related to cancer was estimated to be 13,069 with colorectum, and lung and breast cancer to be the leading causes [3]. Cancer results from transforming normal cells into tumor cells in a multi-stage process that usually progresses from a precancerous lesion to a malignant tumor. There are some risk factors of cancer, including tobacco and alcohol use, unhealthy diets, physical inactivity, viral infections, bacterial infections, urban air pollution, and ionizing radiation [2]. Moreover, some chronic infections can be considered as risk factors for cancer as 13% of cancers diagnosed in 2018 globally were associated with carcinogenic infections [4].

By 2030, it is estimated that the number of cancer cases worldwide will increase to 21.4 million deaths due to changes in population demographics [5]. However, improved survival can be achieved through cancer prevention and early detection strategies. As a result, it is assumed that the new generation may have lower cancer rates in adulthood than in previous generations [6, 7]. Achieving this goal requires a more interdisciplinary and multifaceted approach [6, 7]. Many public and clinical health efforts are required to build a sustainable infrastructure to disseminate preventive measures for cancer control [3, 5, 6].

Screening is a cancer prevention measure for groups who are asymptomatic, and diagnosis is a medical test for groups who are symptomatic [2, 6]. By the time the cancer symptoms appear, the cancer may have grown and spread; making it harder to treat or cure [8–11]. Therefore, screening is vital for easier treat or cure. Moreover, screening can reduce the burden of cancer, and it is considered to be cost-effective. Screening is recommended in many countries [12]. Presently, there are only effective screening recommendations from the American Cancer Society (ACS) and the US Preventive Services Task Force (USPSTF) for six cancer types: breast, cervix, colon, rectum, lung, and prostate [13]. These recommendations vary based on a person’s age and risk factors [13].

In the United States, there was an annual 3.3% decline in cancer incidence among individuals in screen age (65 years and older) during 2000s [14]. However, the incidence of cancer was increased annually by 1 and 2% for individuals between 50 and 64 years and for younger than 50 years old, respectively. Furthermore, mortality rate caused by colorectal cancer (CRC) during 2008 through 2017 was declined by 3% annually in 65 years and older individuals [14].

According to the National Health Interview Survey, the prevalence of colonoscopy for colorectal cancer (CRC) screening among US adults was 62.2% in 2015 [15]. For breast cancer screening, there was only 50.2% female did the mammogram [15]. However, only 6.7% of men aged 50 years or older did the Prostate-Specific Antigen (PSA) testing for prostate cancer screening [15]. However, the data on rate of cancer screening in Saudi Arabia were extremely limited. A study by Almadi et al*.* found that the uptake rate of CRC screening was only around 15% [16]. Breast cancer screening rate in Saudi Arabia reported by Alam. A.A highlighted that despite the knowledge about breast cancer screening among the participants, 61% had information on mammography, and only 18% of the participants did mammography [17].

Recognizing the vital role of health care providers (HCPs) as the most valuable resource for health is fundamental to maintaining public health. At the same time, HCPs must be in good health to treat and care for the patients [8–11]. The medical profession perpetuates the image of the exemplary clinician committed to his/her work, willing to sacrifice everything for medicine—a goal that runs counter to optimal physical and mental well-being; leading to negative impact on their health over time. Nonetheless, while HCPs are constantly exposed to a wide variety of health and safety hazards while maintaining the general population’s health, they do not have lower chance of non-communicable diseases, such as cancer [8, 9].

While considerable research is being done on the overall incidence and prevalence of cancer in Saudi Arabia, there is a lack of data on health care providers' perceptions and attitudes regarding their decision to be screened for cancer. Therefore, this study aimed to assess perceptions and personal attitudes of HCPs regarding their decision to get themselves screened for cancer in the Eastern Province of Saudi Arabia.

## Methods

### Design

A cross-sectional multicenter study was conducted in 2020 at three tertiary hospitals in the Eastern Province of Saudi Arabia; Almoosa Specialist Hospital in Al-Ahsa, tertiary private hospital and National Guard Hospital in Al-Ahsa and in Dammam, governmental hospitals. An Institutional Review Board (IRB) approval was obtained from all the three hospitals, and the study was conducted in accordance with the Declaration of Helsinki. Informed consent was obtained from participants before data collection. The study used a non-probability convenience sample, where all respondents from the three study sites were enrolled consecutively. Eligibility criteria include all HCPs in the three above-mentioned hospitals.

### Sample Size

The sample size was estimated using G*Power3 and based on multiple linear regression using the independent two-tailed *t* test, confidence level of 95%, margin rate of error at 5%, power of 80.0%, medium effect size of 0.40 (determined based on the review of current literature), and a 10% increase to address the non-response rate. The minimum required sample size for this study was 310 subjects.

### Data Collection Tool and Validation

The outcome variable in the current study was the attitude and the perception of HCPs toward the importance of cancer screening. A self-administered pre-structured questionnaire was used to collect data in the present study. The questionnaire consisted of questions on socio-demographics, awareness, and perception of the importance of different cancer screening tests including colonoscopy, PSA, and mammogram. The questions were straightforward, concise, unambiguous, and in logical order. The data collection form was filled directly by respondents. The study did not apply base-line comparison for the two main groups of the HCPs; nurses and physicians.

Before main data collection, a pilot study was conducted among 21 HCPs from the three hospitals for validation purpose. The survey also underwent a statistical assessment for internal consistency, showing a Cronbach alpha value of 84%.

### Statistical Analysis

The collected data were checked for accuracy and completeness before statistical analysis. The Statistical Package for Social Sciences (SPSS version 25) was used for data analysis. A group of descriptive and inferential statistical tests were performed. In the descriptive analysis, socio-demographic variables were analyzed and presented as frequencies and means (M) ± standard deviation (SD). The Chi-square test was applied for inferential statistics. Differences and associations were considered statistically significant if the two-tailed *p* value had a score of ≤ 0.05.

## Results

Out of 900 HCPs who received the questionnaire, 372 HCPs responded. The mean age of the study participants was 34.1 ± 7.1 years. Around 40% of the participants were from a private tertiary hospital and two-thirds were from two governmental hospitals. Nurses constituted the majority of the study participants; around two-thirds. The majority of participants were females; 67.5, and 75.5% of the participants were non-Saudi citizens (Table 1).

**Table 1:** Baseline socio-demographic characteristics (*n* = 372)

Characteristics	*N* (%)
Hospital site	
Private tertiary hospital Al-Ahsa city	142 (38.2)
Governmental tertiary hospital Al-Ahsa city	160 (43.0)
Governmental tertiary hospital Dammam city	70 (18.8)
Gender	
Male	121 (32.5%)
Female	251 (67.5%)
Nationality	
Saudi	91 (24.5%)
Non-Saudi	281 (75.5%)
Marital status	
Single	130 (34.9%)
Married	226 (60.8%)
Divorced or widowed	16 (4.3%)
Have kids	
Yes	214 (57.5)
No	158 (42.5)
Monthly income	
< 5000	109 (28.0)
5000–10,000	124 (33.3)
> 10,000	134 (38.7)
Professional group	
Physician	125 (33.6%)
Nurse	247 (66.4%)
Educational background	
Bachelor	267 (71.8%)
Master and PhD	47 (12.6%)
Diploma	35 (9.4%)
Fellowship	23 (6.2%)
Age categories	
Lower than 40 years	302 (81.2)
40—44 years	34 (9.1)
45—54 years	31 (8.3)
55 years and above	5 (1.3)
Mean ± SD	34.1 ± 7.1 years

Regardless of gender, profession, or age, the overall rate of belief in the protective value of regular cancer screening was high; 91.4%. The percentage of participants who have done colonoscopy for their selves was 3.5. However, the percentage of female participants who have done mammography screening was only 15.9, and the percentage of male who got themselves screened for PSA was 12.4 (Table 2). Using univariate analysis for cancer screening for each group, categories showed that all study participants above 54 years old have not done colonoscopy, while 19.4% of the study participants aged between 45 and 54 years have been screened. For mammography, the two female participants aged above 54 years have not done it, while 68.4% of the participated female aged between 45 and 54 years have been screened for breast cancer. For PSA, majority of the male aged above 54 years have done PSA, while 99% of study participants aged below 45 years did not do the PSA testing for prostate cancer (Table 3).

**Table 2:** Perception of health care providers on cancer screening (*n* = 372)

Characteristics	*N* (%)
Do you believe in the importance of cancer screening?	
Yes	340 (91.4)
No	32 (8.6)
Have you done colonoscopy for yourself?	
Yes	13 (3.5)
No	359 (96.5)
Have you done mammography yourself?	
Yes	40 (15.9%) of female
Have you done PSA yourself?	
Yes	15 (12.4) of male

**Table 3:** Univariate analysis for cancer screening per age categories (*n* = 372)

Type of the screening test	Screened (*N*%)	Not screened (*N*%)	*p* Value
Mammography for 251 females			0.0001
Below 45 years	27 (11.7)	203 (88.3)	
45–54 years	13 (68.4)	6 (31.6)	
Above 54 years	0 (0.0)	2 (100.0)	
Colonoscopy for total 372			0.0001
Below 45 years	7 (2.1)	329 (97.9)	
45–54 years	6 (19.4)	25 (80.6)	
Above 54 years	0 (0.0)	5 (100.0)	
PSA for 121 males			0.0001
Below 45 years	1 (1.0)	95 (99.0)	
45–54 years	6 (35.3)	11 (64.7)	
Above 54 years	5 (62.5)	3 (37.5)	

**Fig. 1:**
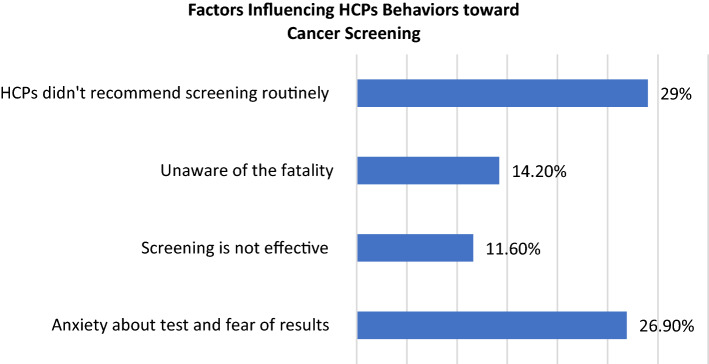
Factors influencing cancer screening choice of HCPs

**Fig. 2:**
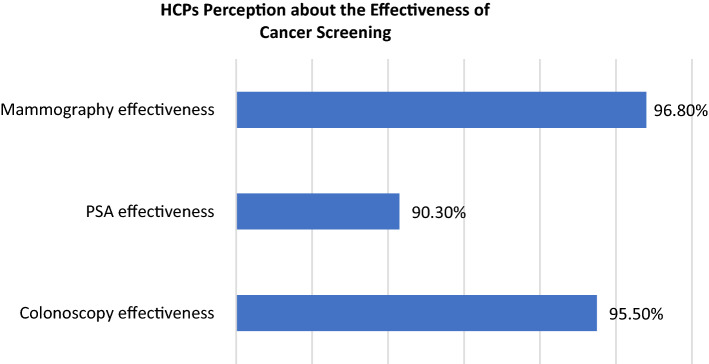
Effectiveness of cancer screening

Regarding factors affecting screening perception of the respondents, 29% of the study participants stated that the HCPs did not often recommend it to their patients. The second most common factor was anxiety about test and fear of result; 26.9% (Fig. 1). On the other hand, questions on the perceptions toward the effectiveness of screening showed that HCPs perceived that the mammography was considered as the most effective; 96.80%, followed by colonoscopy, and PSA (Fig. 2).

## Discussion

The primary objective of this study was to assess the perceptions and personal attitudes of HCPs regarding their decision to screen themselves for cancer in the Eastern Province of Saudi Arabia. The findings showed that most respondents irrespective of their profession, gender, and age believed in the importance of cancer screening. However, participants’ beliefs were not reflected in their behavior toward screening for themselves as out of the 372 HCPs participated in the study, only 3.5% of them have done colonoscopy; agreeing with a study finding conducted in Saudi Arabia. Shaheen et al. (2021) reported that HCPs in Saudi Arabia showed poor knowledge of screening guidelines and that participants did not adhere to screening tests on their own, especially for breast, cervical, and colon cancers for older age groups or with a positive family history cancer in the family [18].

According to the American Cancer Society (ACS) guidelines, it is recommended for women aged between 45 and 54 to get mammograms every year [19]. For CRC, it is recommended to start regular screening at the age of 45 years for people at average risk [19]. Men at age of 50 years should be screened for prostate cancer by PSA testing [19]. A study in Saudi Arabia conducted by El Bcheraoui et al. (2015) reported that 92% of women aged 50 years and older never had a mammogram [20]. However, our study showed that 84% of all female participants have never done mammogram. However, the mean age for all study participants was 34.1 ± 7.1 years in which mammogram is not indicated in this age group. On the other hand, for age group between 45 and 54 years, 68.4% of the female have done mammogram which may reflect a better awareness. This finding may be explained by the nature of the participant profession as HCPs may have a better knowledge regarding health issues.

The most common reason perceived by the respondents that influenced the decision to get themselves screened for cancer was that HCPs did not educate them regarding screening. This finding is consistent with the study by Hweissa et al. (2016) and a study by Mosli et al. (2017). Hweissa et al. (2016) reported that while HCPs can influence women’s decision about cervical cancer screening, the lack of screening recommendations may be one of the barriers affecting women’s participation in screening programs [21]. HCPs are one of the reliable information sources for patients regarding screening recommendations. Therefore, HCPs have the responsibility to communicate effectively with their patients event if the patients are HCPs. Additionally, Mosli et al. (2017) reported that a significant proportion of primary care providers do not adhere to screening guidelines for CRC, despite the widespread belief that screening is effective [22]. A randomized trial conducted in two community health center practices between January 2014 and March 2016 showed higher CRC screening among patients receiving a decision aid and navigation support compared to patients receiving usual care [23]. Health professionals should provide guidance to adults about the benefits, limitations, and potential burdens associated with screening test options and assist them in making a choice and completing screening [21].

The second most common reason for participants to influence their decision regarding screening was the concern and anxiety about getting positive results. However, findings from the literature showed that not all positive results lead to cancer identification. For instance, 12 abnormal mammograms and three or more biopsies are performed before one case of breast cancer is found [12]. Moreover, Thus, similar to the general population, psychological factors, including anxiety and fear, will contribute to poor screening among HCP; on the other hand, improved knowledge and information about cancer screening is key to increased participation and adherence. Therefore, educational programs are recommended, particularly among HCPs as they can further educate their patients about cancer risk factors, the importance of cancer screening, and distinctive features of screening modalities [9].

The results of this study should be interpreted in light of its limitations. The study used an observational design that is inherently open to controversy and has the risk of containing biases. The study covers only the Eastern Province of Saudi Arabia. Therefore, more research should be carried out in all Saudi Arabia regions to gain a broader picture and develop training programs for health care providers to enrich knowledge about cancer screening, its advantages, and disadvantages.

## Conclusion

Findings of the current research and existing evidence specifically for the Saudi community indicated a need to raise awareness, emphasizing the role of HCPs in motivating themselves, their families, and their patients to implement various cancer screening programs. This can be achieved through further research about the additional education or training needed for HCPs, which could positively influence their attitude toward cancer screening, making the procedure part of their own routine medical examination.

## Data Availability

Data used and analyzed in this study will be promptly available for the publisher upon request.

## Abbreviations

PSA: Prostate-specific antigen
HCP: Healthcare providers
HPV: Human papilloma virus
HIV: Human immunodeficiency virus
NCD: Non-communicable diseases
SPSS: Statistical Package for Social Sciences
CRC: Colorectal cancer
