# Correction to: Attitudes and Practice of Health Care Providers Toward Cancer Screening: A Cross-sectional Multicenter Study, Saudi Arabia

**DOI:** 10.1007/s44197-022-00074-0

**Published:** 2022-11-18

**Authors:** Gasmelseed Y. Ahmed, Abbas Al Mutair, Shahinaz Bashir, Rommel Acunin, Nora Al Aljabr, Rasha Alnumari, Ghina Alarab, Siddig Mohamed Hussein, Chandni Saha, Lamiaa H. Al-Jamea, Alexander Woodman, Eman Almusalami

**Affiliations:** 1 College of Medicine and Health Sciences, Almanagil University, Almanagil, Sudan; 2 Research Center Almoosa specialist hospital, Alahsa, Saudi Arabia; 3 School of Nursing, Wollongong University, Wollongong, Australia; 4 Almoosa College of Health Sciences, Alahsa, Saudi Arabia; 5 Nursing Department, Princess Nourah Bint Abdul Rahman University, Riyadh, Saudi Arabia; 6 King Abdullah International Medical Research Center (KAIMRC), Al-Ahsa, Saudi Arabia; 7 King Abdullah International Medical Research Center (KAIMRC), Dammam, Saudi Arabia; 8 Almoosa Specialist Hospital, Alahsa, Saudi Arabia; 9 Vice Deanship of Postgraduate studies and Research, Prince Sultan Military College of Health Sciences, Dhahran, Saudi Arabia

## Correction to: Journal of Epidemiology and Global Health 10.1007/s44197-022-00056-2

In this article the author name Alexander Woodman was incorrectly written as Alxeander Woodman. The original article has been corrected.

